# A deep insight into the sialotranscriptome of the mosquito, *Psorophora albipes*

**DOI:** 10.1186/1471-2164-14-875

**Published:** 2013-12-13

**Authors:** Andrezza C Chagas, Eric Calvo, Claudia M Rios-Velásquez, Felipe AC Pessoa, Jansen F Medeiros, José MC Ribeiro

**Affiliations:** 1 Laboratory of Malaria and Vector Research, National Institute of Allergy and Infectious Diseases, National Institutes of Health, Rockville, MD, USA; 2 Biodiversity Laboratory, Instituto Leônidas e Maria Deane/Fiocruz, Manaus, Amazonas, Brazil; 3 Entomology Laboratory, Fiocruz Rondônia, Porto Velho, Rondônia, Brazil

## Abstract

**Background:**

*Psorophora* mosquitoes are exclusively found in the Americas and have been associated with transmission of encephalitis and West Nile fever viruses, among other arboviruses. Mosquito salivary glands represent the final route of differentiation and transmission of many parasites. They also secrete molecules with powerful pharmacologic actions that modulate host hemostasis, inflammation, and immune response. Here, we employed next generation sequencing and proteome approaches to investigate for the first time the salivary composition of a mosquito member of the *Psorophora* genus. We additionally discuss the evolutionary position of this mosquito genus into the Culicidae family by comparing the identity of its secreted salivary compounds to other mosquito salivary proteins identified so far.

**Results:**

Illumina sequencing resulted in 13,535,229 sequence reads, which were assembled into 3,247 contigs. All families were classified according to their in silico-predicted function/ activity. Annotation of these sequences allowed classification of their products into 83 salivary protein families, twenty (24.39%) of which were confirmed by our subsequent proteome analysis. Two protein families were deorphanized from *Aedes* and one from *Ochlerotatus,* while four protein families were described as novel to *Psorophora* genus because they had no match with any other known mosquito salivary sequence. Several protein families described as exclusive to Culicines were present in *Psorophora* mosquitoes, while we did not identify any member of the protein families already known as unique to Anophelines. Also, the *Psorophora* salivary proteins had better identity to homologs in *Aedes* (69.23%)*,* followed by *Ochlerotatus* (8.15%)*, Culex* (6.52%), and *Anopheles* (4.66%), respectively.

**Conclusions:**

This is the first sialome (from the Greek sialo = saliva) catalog of salivary proteins from a *Psorophora* mosquito, which may be useful for better understanding the lifecycle of this mosquito and the role of its salivary secretion in arboviral transmission.

## Background

*Psorophora* mosquitos—commonly known as “giant mosquitoes”—belong to the subfamily Culicinae, which includes many genera with epidemiologic importance to humans and animals such as *Aedes*, *Ochlerotatus*, *Haemagogus,* and *Culex*. Notably, members of the *Psorophora* genus are found only in the New World. *Psorophora* mosquitoes are opportunistic, having mammals and birds as the main hosts of their blood-feeding [1,2]. *Psorophora* females have been associated with transmission of equine encephalitis virus, West Nile fever virus, and other arboviruses [3-9].

The phylogeny of mosquitoes includes three subfamilies within the Culicidae: Anophelinae, Culicinae, and Toxorhynchitinae. Studies based on the morphology, behavior, biogeographic distribution, and life-history suggest the Anophelinae subfamily as monophyletic and basal into the Culicidae family. On the other hand, the Culicinae subfamily includes the majority of remaining mosquito genera distributed into ten tribes. *Psorophora* mosquitoes share the tribe Aedini together with *Aedes*, *Ochlerotatus,* and other mosquito genera, while *Culex* mosquitoes belong to the Culicini tribe. Previous studies have supported the genera from the tribe Culicini as basal to genera of the tribe Aedini [10]. These results are in agreement with the phylogeny proposed by Besansky and Fahey [11]. The *Psorophora* genus contains 48 species divided into three subgenera: *Grabhamia* (15 species), *Janthinosoma* (23 species), and *Psorophora* (10 species) [12]. Recently, morphologic and molecular studies have supported *Psorophora* as a sister group with *Aedes/Ochlerotatus*[13-15]. In contrast, studies using 18S rDNA sequence have suggested *Psorophora* species as a sister group to *Culex* and/or to *Aedes*/*Ochlerotatus* species [12,16].

The salivary glands (SGs) of hematophagous insects secrete a cocktail of biochemically active compounds [17] that interacts with hemostasis [18-21], immunity, and inflammation of their hosts [22,23]. Perhaps because of the continuous contact of mosquito salivary proteins with host immunity, salivary proteins are at a fast pace of evolution and divergence, even in closely related species [24]. In the past decade, the continuous advances in the fields of transcriptome and proteome analysis led to the development of high-throughput sialotranscriptome studies (from the Greek *sialo* = saliva) [23,25]. These studies resulted in a large database of secreted salivary proteins from different blood-feeding arthropod families including members of the Culicidae family.

All mosquito sialotranscriptome studies so far have targeted members of the *Aedes, Ochlerotatus, Anopheles,* and *Culex* genera [24], which are important vectors of human and animal diseases*.* Although some *Psorophora* species are known to be vectors of several arboviruses, the molecular composition of their salivary secretion remains unknown. Our primary aim was to investigate the salivary transcriptome and proteome of a member of the *Psorophora* genus (*Psorophora albipes*) to ultimately better understand the evolution of SG composition within the Culicidae family. In addition, our work makes available the first platform of salivary proteins from this mosquito genus, relevant for improving our understanding of mosquito evolution, the evolving risks in public health due to the recent expansions of *Psorophora* mosquitoes to the North, and for development of exposure markers to mosquito bites and to vector-borne diseases transmitted by mosquitoes.

## Methods

### Mosquitoes

*Psorophora* mosquitoes were collected in fragments of unflooded rain forest in Manacapuru municipality, Amazonas state, Brazil, using modified CDC traps. The mosquitoes were maintained with water and sugar solution and transported to Biodiversity Laboratory of Leônidas and Maria Deane Institute (Fiocruz/Manaus). The mosquitoes were identified using the taxonomic keys proposed by Forattini [12] and Consoll and Lourenco de Oliveira [26].

### Dissection and RNA extraction

SGs from *P. albipes* (50 pairs) were dissected in 150 mM sodium chloride pH 7.4 and immediately transferred to 50 μl RNAlater® solution and maintained at 4°C until the RNA extraction. SG RNA was extracted and isolated using the Micro-FastTrack™ mRNA isolation kit (Invitrogen, San Diego, CA) per manufacturer’s instructions. The integrity of the total RNA was checked on a Bioanalyser (Agilent Technologies Inc., Santa Clara, CA).

### Next-Generation Sequencing (NGS) and bioinformatic analysis

The SG library was constructed using the TruSeq RNA sample prep kit, v2 (Illumina Inc., San Diego, CA). The resulting cDNA was fragmented using a Covaris E210™ focused ultrasonicator (Covaris, Woburn, MA). Library amplification was performed using eight cycles to minimize the risk of over-amplification. Sequencing was performed on a HiSeq 2000 (Illumina) with v3 flow cells and sequencing reagents. One lane of the HiSeq machine was used for this and two other libraries, distinguished by bar coding. A total of 135,651,020 sequences of 101 nt in length were obtained. A paired-end protocol was used. Raw data were processed using RTA 1.12.4.2 and CASAVA 1.8.2. mRNA library construction, and sequencing was done by the NIH Intramural Sequencing Center (NISC). Reads were trimmed of low-quality regions (< 10) and were assembled together with the assembly by short sequences (ABySS) software (Genome Sciences Centre, Vancouver, BC, Canada) [27,28] using various kmer (k) values (every even number from 24 to 96). Because the ABySS assembler tends to miss highly expressed transcripts [29], the Trinity assembler [30] was also used. The resulting assemblies were joined by an iterative BLAST and cap3 assembler [31]. Sequence contamination between bar-coded libraries were identified and removed when their sequence identities were over 98%, but their abundance of reads were > 50 fold between libraries. Coding sequences (CDS) were extracted using an automated pipeline, based on similarities to known proteins, or by obtaining CDS containing a signal peptide [32]. Coding and their protein sequences were mapped into a hyperlinked Excel spreadsheet (presented as Additional file 1, and also located at http://exon.niaid.nih.gov/transcriptome/Psorophora_albipes/Pso-s2-web.xlsx.). Signal peptides, transmembrane domains, furin cleavage sites, and mucin-type glycosylation were determined with software from the Center for Biological Sequence Analysis (Technical University of Denmark, Lyngby, Denmark) [32-35]. Reads were mapped into the contigs using blastn [36] with a word size of 25, masking homonucleotide decamers and allowing mapping to up to three different CDS if the BLAST results had the same score values. Mapping of the reads was also included in the Excel spreadsheet. Automated annotation of proteins was based on a vocabulary of nearly 250 words found in matches to various databases—including Swissprot, Gene Ontology, KOG, PFAM, and SMART, and a subset of the non-redundant protein database of the NCBI containing proteins from vertebrates. Further manual annotation was done as required. Detailed bioinformatics analysis of our pipeline can be found in our previous publication [31]. Sequence alignments were done with the ClustalX software package [37]. Phylogenetic analysis and statistical neighbor-joining bootstrap tests of the phylogenies were done with the Mega package [38]. Blast score ratios were done as indicated previously [39]. For visualization of synonymous and non-synonymous sites within coding sequences, the tool BWA aln [40] was used to map the reads to the CDS, producing SAI files that were joined by BWA sampe module, converted to BAM format, and sorted. The sequence alignment/map tools (samtools) package [41] was used to do the mpileup of the reads (samtools mpileup), and the binary call format tools (bcftools) program from the same package was used to make the final vcf file containing the single-nucleotide polymorphic (SNP) sites, which were only taken if the site coverage was at least 100 (-D100), the quality was 13 or better and the SNP frequency was 5 or higher (default). Determination of whether the SNPs lead to a synonymous or non-synonymous codon change was achieved by a program written in Visual Basic by JMCR, the results of which are mapped into the Excel spreadsheet and color visualized in hyperlinked rtf files within Additional file 1.

### Proteome analysis

Fifty SG pairs from female *P. albipes* were used in the proteome analysis. Briefly, the glands were sonicated and the supernatant was boiled for 10 min in reducing Laemmli gel-loading buffer and subsequently resolved on a NuPAGE 4-12% Bis-Tris precast gradient gel. Proteins were visualized with SimplyBlue stain (Invitrogen). The gel was arbitrary sliced into 19 individual sections (coded as F1–19) that were destained and digested overnight with trypsin at 37°C. ZipTips® (Millipore, Belford, MA) were used to extract and desalt the peptides, which were resuspended in 0.1% TFA before mass spectrometry analysis (MS).

Nanoflow reverse-phase liquid chromatography coupled with tandem MS (MS/MS) was performed as described [42]. We obtained a database of the tryptic peptides identified by MS as a final product. This was used to search for matches from our transcriptome database of *P. albipes*. Additional details about the proteome procedure and analysis can be found in the methodology described in Chagas et al. [42].

## Results and discussion

### Exploring the sialotranscriptome of a *Psorophora* mosquito

Assembly of 135,651,020 reads into 43,466 contigs (see assembly details in Additional file 2) allowed the extraction of 3,247 CDS (which mapped 13,535,229 reads) which in turn were classified according to their primary sequence (presence of homology to an already described sequence) into three categories: *i*) transcripts encoding for secreted (S) proteins, *ii*) transcripts encoding for housekeeping (H) proteins, and *iii*) transcripts encoding for proteins of unknown (U) function that lack homology with any functionally characterized protein from another organism (Table 1). Notice that these 3,247 CDS contain 485 similar CDS divergent by a few amino acids, which can be verified in the clusterization column grouping protein sequences with 95% similarity on 50% of their lengths. These may represent allele products, recent gene duplications, or sequencing/assembly errors.

**Table 1:** **Functional classification of transcripts from salivary glands of the mosquito *Psorophora albipes***

**Class**	**Number of coding sequences**	**% of CDS**	**Number of reads**	**% of total reads**
Secreted	802	24.70	7,537,805	55.69
Housekeeping	1,973	60.76	5,473,151	40.44
Transposable element	38	1.17	85,213	0.63
Unknown product	434	13.37	439,060	3.24
Total	3,247	100.00	13,535,229	100.00

After annotation, 7,537,805 reads (55.69% of the reads mapped to CDS) were classified as originating from transcripts encoding putative S proteins, and these were assembled into 802 contigs (24.70% of the total contigs) (Table 1). Signal peptide was detected in these contig sequences, suggesting that these contigs encode for proteins secreted in the saliva. In addition, 5,473,151 transcript reads (40.44% of the total reads) mapped to transcripts encoding H proteins, which were assembled into 1,973 contigs (60.76% of the total contigs). Another 85,213 reads (0.63% of total reads) correspond to transposable elements, and 439,060 reads (3.24% of total reads) were classified as originating from transcripts that encode for U products (Table 1).

The sequences encoding for H proteins were further classified into 26 subgroups according to their predicted function or membership to previously described protein families (Table 2). The potentially highly expressed H proteins include those associated with protein synthesis machinery (14.92% of the reads classified as H products), signal transduction proteins (5.18% of the total reads), unknown conserved—which represent highly conserved proteins of unknown function most likely related with cellular function (3.25% of the total reads), transporters and channels (3.18% of total reads), and proteins with a potential role in lipid metabolism (2.84% of the total reads). Because SGs are specialized in secretion, high expression of transcripts encoding for constituents of protein synthesis machinery and energy metabolism is commonly observed in similar analyses of blood-feeding arthropod sialotranscriptomes. Here energy metabolism represents only 0.86% of the total transcript reads encoding for H products.

**Table 2:** **Functional classification of the housekeeping products expressed in the female *Psorophora albipes *** **salivary glands**

**Housekeeping**	**Number of contigs**	**Number of reads**	**% Reads**
Protein synthesis machinery	132	1,711,561	14.92
Signal transduction	283	594,284	5.18
Unknown conserved	265	372,425	3.25
Transporters and channels	192	364,580	3.18
Lipid metabolism	90	326,204	2.84
Transcription machinery	122	292,742	2.55
Protein export	136	236,848	2.06
Cytoskeletal proteins	100	202,027	1.76
Protein modification	68	185,852	1.62
Extracellular matrix	65	159,217	1.39
Storage	14	149,093	1.30
Unknown conserved with transmembrane domains	51	140,420	1.22
Energy metabolism	76	98,777	0.86
Carbohydrate metabolism	55	96,997	0.85
Proteases	45	94,848	0.83
Amino acid metabolism	28	83,082	0.72
Proteasome machinery	85	76,054	0.66
Intermediary metabolism	10	60,190	0.52
Native immunity	21	27,736	0.51
Signal transduction - apoptosis	37	50,036	0.44
Transcription factor	26	33,506	0.29
Nucleotide metabolism	15	33,498	0.29
Nuclear regulation	22	31,366	0.27
Oxidant metabolism/Detoxification	19	22,649	0.20
Nuclear export	6	16,187	0.14
Detoxification	10	12,972	0.11
Total	1973	5,473,151	47.71

The putative S proteins were further divided into 16 general categories (Table 3), several of which were abundantly expressed in *P. albipes* SGs at the transcriptome level. Their members had a classic secretion signal: mucin I mosquito family (24.77% of total reads classified as S products), similar to OT-19 containing HH repeats family (10.25% of total reads), glycosidases (9.05% of total reads), HHH peptide family (peptides containing a His triad) (7.65% of total reads), 30.5-kDa family (4.35% of total reads), long-D7 mosquito family (3.95% of total reads), Antigen-5 family (3.42% of total reads), Aegyptin family (2.62% of total reads), Serpin family (2.24% of total reads), Culicine short-D7 protein family (2.08% of total reads), *Aedes* 5-kDa family (1.67% of total reads), 41-kDa canonical family (1.45% of total reads), and Hyp8.2 Culicine family (1.30% of total reads) (Table 3). Additionally, eight novel protein families were described in *P. albipes* with either no significant matches to any sequence deposited in the NCBI database, or matching mosquito hypothetical proteins not previously described in sialotranscriptomes; these were named Psor 4.7 kDa, Psor 4.2 kDa, Psor 12 kDa, Psor 6.3 kDa, Psor 4.01 kDa ultrashort-D7 family, Psor 12.8 kDa novel mosquito peptide family, Psor 4.69 kDa weakly similar to *Aedes*, and Psor 20.44 kDa weakly similar to Culicine. These new protein families account for 1% of all the transcripts reads of *P. albipes* SG transcriptome. A summary and details related to the transcript annotation encoding for S proteins can be found in Table 3 and in Additional file 1.

**Table 3:** **Functional classification of transcripts coding for putative secreted proteins in female *Psorophora albipes *** **salivary glands**

**Secreted**	**Number of**		**% Secreted reads**
	**Contigs**	**Reads**	
**Enzymes**			
Glycosidases	7	681,837	9.05
5′ nucleotides/Apyrase family	6	41,550	0.55
Serine proteases	5	40,574	0.54
Cathepsins	3	36,397	0.48
Serine-type carboxypeptidases	2	20,779	0.28
Mosquito lipases	1	9,608	0.13
Alkaline phosphatases	2	8,087	0.11
Adenosine deaminase family	1	6,885	0.09
Endonucleases	4	6,281	0.08
Ribonucleases	3	2,417	0.03
Destabilase family	1	842	0.01
Hyaluronidases	1	372	0.00
**Ubiquitous protease inhibitor domains**			
Serpin family	15	169,094	2.24
Kazal domain-containing peptides	13	46,712	0.62
TIL domain family found in mosquitoes	8	38,268	0.51
Metalloproteinase inhibitors	1	2,029	0.03
Schistocerca protease inhibitor	1	2,023	0.03
Cystatins—may be housekeeping	1	175	0.00
**Immunity-related proteins**			
Fred/Ficolin domain-containing proteins	9	65,681	0.87
Lysozymes	7	59,845	0.79
C type lectins	7	58,303	0.77
Peptidoglycan recognition domains	7	16,505	0.22
Gram-negative binding proteins	1	5,968	0.08
ML domains	3	4,480	0.06
Gambicin	2	4,360	0.06
Leucine-rich proteins	1	1,249	0.02
Cecropins	1	277	0.00
Defensin	1	275	0.00
Galectin—maybe housekeeping	1	173	0.00
**Mucins**			
Mucin I mosquito family	22	1,866,771	24.77
gSG5 mucin protein family	8	60,819	0.81
Aedes-specific mucins	7	30,550	0.41
Other mucins	8	24,225	0.32
Virus-induced mucins	1	11,007	0.15
SG3 mucin family	2	2,197	0.03
Peritrophin/chitin binding	3	1,587	0.02
*Simulium* mucins	3	1,320	0.02
**OBP superfamily**			
Long-D7 mosquito family	26	297,507	3.95
Culicine short-D7 proteins	23	156,429	2.08
Salivary mosquito OBP	2	1,028	0.01
**Yellow Phlebotomine family**	2	7,763	0.10
**Ubiquitous protein families existing outside Nematocera, function unknown**			
Antigen-5 family	13	257,852	3.42
*Aedes* hypothetical secreted conserved proteins	3	5,605	0.07
12- to 14-kDa mosquito family similar to *Drosophila* proteins	4	3,609	0.05
15- to 17-kDa insect family	1	816	0.01
*Culex/Drosophila* WAP subfamily	1	283	0.00
**Protein families exclusive of bloodsucking Nematocera**			
30 kDa/Aegyptin family - Mosquitoes and black flies	5	197,279	2.62
Canonical	8	109,171	1.45
Mucin II mosquito family	2	9,705	0.13
**Protein families specific to mosquitoes**			
HHH peptide family	18	576,548	7.65
30.5-kDa protein	4	327,913	4.35
hyp8.2 Culicine family	6	98,361	1.30
9.7-kDa family	17	63,969	0.85
*Aedes* W-rich peptides	1	51,783	0.69
*Aedes* 6.5–8.5 protein family	3	43,900	0.58
*Aedes* 62-kDa family	6	41,700	0.55
Basic tail mosquito family	6	32,687	0.43
34-kDa *Aedes* family	8	30,082	0.40
*Aedes/Anopheles darlingi* 14–15 family	6	25,350	0.34
GQ-rich Culicine family	3	8,203	0.11
23.5 kDa Culicine family	4	8,099	0.11
gSG8 family	1	3,237	0.04
Hyp6.2 family	3	2,291	0.03
*Culex* WRP/16-kDa family	2	2,138	0.03
Salivary protein 16 family	2	1,778	0.02
HHH family 2	3	1,432	0.02
Anopheline SG1 family	1	273	0.00
**Protein families specific to black flies**			
*Simulium* disintegrin similar to phenoloxidase inhibitor peptides	1	15,887	0.21
H-rich, acidic proteins of *Simulium*	1	861	0.01
**Salivary-orphan proteins of conserved secreted families**	9	11,269	0.15
**Deorphanized proteins**			
Similar to OT-19—contains HH repeats	16	772,288	10.25
*Aedes* 5-kDa family	7	125,986	1.67
*Aedes* 7-kDa family	4	2,378	0.03
**Families not reported on Nematocera - sialome review**			
Lipocalins	5	23,805	0.32
SGS family	4	4,869	0.06
**Novel families**			
Pso 4.7 kDa	6	47,608	0.63
Pso 4.01 kDa ultrashort-D7 family	3	11,185	0.15
Pso 4.2 kDa	12	4,201	0.06
Pso 6.3 kDa	3	3,398	0.05
Pso 12 kDa	3	1,289	0.02
Pso 12.8 kDa—novel mosquito peptide family	2	1,217	0.02
Pso 20.44 kDa—unique to Culicine	1	290	0.00
Pso 4.69 kDa—unique to *Aedes*	1	103	0.00
**Other putative secreted proteins**	**371**	**814,858**	10.81
**Total secreted**	**802**	**7,537,805**	

### Proteomics analysis of *P. albipes* SGs

**Figure 1:**
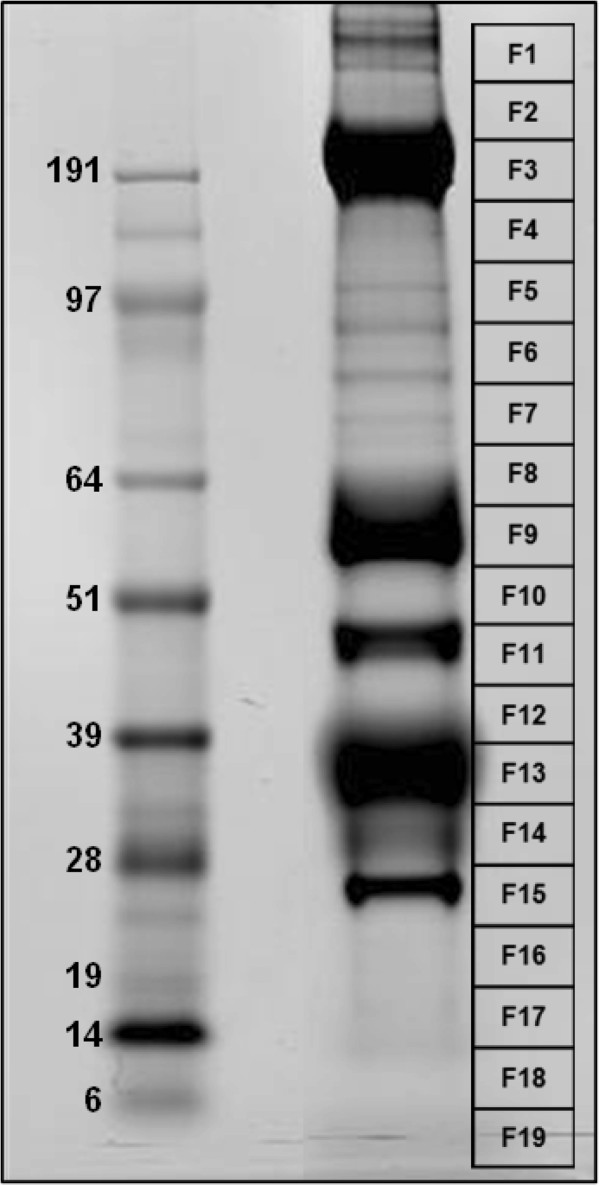
**Salivary gland proteins from the mosquito *Psorophora albipes.*** The left gel lane shows the protein standards with their molecular weights (kDa). The right gel lane shows the *P. albipes* salivary proteins (Coomassie stained). The grid at the right (F1–F19) shows the area of the gel slices submitted for tryptic digest and tandem mass spectrometry identification.

We employed a proteomics analysis to investigate protein expression in SGs of *P. albipes*. After Coomassie staining, five bands were revealed as strongly stained at approximate molecular weight (MW) near 191 kDa, 64 to 51 kDa, between 51 to 39 kDa, between 39 to 28 kDa, and one last band with an estimated MW of 28 kDa. Other bands with lesser stain intensity were also revealed in the gel (Figure 1). The NuPage gel was arbitrary cut into 19 fractions and submitted to MS/MS analysis. Contigs showing up two or more tryptic peptides were identified by using the *P. albipes* transcriptome database. Table 4 presents the details of all secreted contigs identified in the *P. albipes* SG proteome.

**Table 4:** **Putative secreted proteins identified in the sialotranscriptome of *Psorophora albipes *** **and confirmed by our proteomic studies**

**Description**	Protein name | Fraction → Number of Tryptic peptides*
5′ nucleotidase/apyrase	Psor-13556|F11→8, Psor-17515|F11→8, Psor-22761|F11→8, Psor-17516|F11→7, Psor-12600|F11→5, Psor-17320|F11→4
Adenosine deaminase	Psor-13638|F11→163
Endonuclease	Psor-34082|F12→18, Psor-34081|F12→18, Psor-34084|F12→8
Glycosidases	Psor-12511|F9→84
Serpin	Psor-18383|F12→594, Psor-18379|F12→562, Psor-18398|F12→504, Psor-18380|F12→473, Psor-20123|F12→263, Psor-20135|F12→239, Psor-18402|F12→207, Psor-20121|F12→159, Psor-18262|F12→151, Psor-12755|F12→19
Lysozyme	Psor-13510|F19→7, Psor-14008|F19→7, Psor-12935|F19→7, Psor-29999|F19→7
gSG5 mucin	Psor-13808|F7→34, Psor-12515|F7→29, Psor-13816|F7→24, Psor-21012|F7→23, Psor-12520|F7→16, Psor-16372|F14→3
*Aedes*-specific mucin	Psor-24903|F14→8
Long-D7 mosquito	Psor-34191|F15→29, Psor-34198|F15→28, Psor-34194|F15→28, Psor-34190|F15→22, Psor-34202|F15→17, Psor-32651|F15→9, Psor-16545|F14→26, Psor-14130|F14→22, Psor-27735|F14→22, Psor-22249|F14→22, Psor-21941|F14→10, Psor-14244|F14→5, Psor-22962|F14→5, Psor-16516|F14→4, Psor-16517|F14→3, Psor-5239|F14→2
Short-D7 Culicine	Psor-24290|F19→11, Psor-14799|F19→8, Psor-24280|F19→8, Psor-14821|F19→8, Psor-24282|F19→8, Psor-24283|F19→7, Psor-11438|F19→5, Psor-33959|F19→3
Antigen-5	Psor-12020|F16→22, Psor-14194|F16→5, Psor-12762|F15→7, Psor-17302|F15→3
30-kDa/Aegyptin family	Psor-20191|F15→39, Psor-18075|F14→19, Psor-18076|F14→18, Psor-18880|F14→10, Psor-19455|F14→7
Basic tail mosquito	Psor-13880|F19→3
*Aedes* 62-kDa	Psor-23962|F18→7, Psor-23027|F12→4, Psor-18121|F12→2, Psor-15772|F12→2, Psor-15774|F12→2
9.7-kDa family	Psor-19686|F19→5, Psor-19685|F19→4, Psor-19523|F19→3, Psor-12072|F19→3
Hyp8.2 Culicine	Psor-31485|F19→2, Psor-31484|F19→2, Psor-31419|F19→2
30.5-kDa	Psor-14979|F15→4
23.5-kDa	Psor-16925|F17→2
34-kDa Aedes	Psor-15738|F14→2
Psor-4 kDa ultrashort-D7	Psor-9075|F19→2

*Summary in the form of F11 →18∣ F12 →18∣ F13→2∣ indicates that 18 fragments were found in Fraction 11, while 18 and 2 peptides were found in fractions 12 and 13, respectively. This summary included protein identification only when two or more peptide matches to the protein were obtained from the same gel slice.

We confirmed expression of 20 of 83 (24.09%) S protein families described in the sialotranscriptome. The three strongly stained bands of the gel apparently match to: F9 (glycosidase family), F11 (apyrase, adenosine deaminase), F15 (long-D7 mosquito family, 30-kDa Aegyptin family, Antigen-5). To conclude, six of ten protein families described as highly expressed in our *P. albipes* SG transcriptome (glycosidases, 30.5-kDa family, long-D7 mosquito family, 30-kDa Aegyptin-like family, Serpin family, and Culicine D7 mosquito family, respectively) were confirmed to be present in the salivary proteome of *P. albipes* based on our subsequent proteomics analysis. Furthermore, seven families (35% of the total families confirmed by proteome) described in the transcriptome as specific for mosquitoes (basic tail mosquito, *Aedes* 62 kDa, 9.7-kDa family, Hyp8.2 Culicine, 30.5-kDa family, 23.5-kDa family, *Aedes* 34 kDa) were also confirmed by our proteome analysis. Additionally, the proteomics analysis confirmed the presence of the newly described protein family named as “Psor-4 kDa ultrashort-D7 family–Contig Psor-9075.” More details about contigs/families found in the proteome of *Psorophora* can be seen in Figure 1 and Table 4. Tryptic peptides were assigned to several contigs encoding for H proteins (Additional file 1) such as a *P. albipes* Sphingomyelin phosphodiesterase that shows 55% amino acid identity to the homolog/ortholog from *Culex quinquefasciatus*. Previous proteomic studies using mosquito SGs identified some abundant protein families in *Aedes aegypti* such as long-D7 protein, adenosine deaminase, serpin, and 30-kDa Aegyptin [43]. Members of all these families were similarly identified in our *P. albipes* proteome. Additionally, members of the two mosquito-specific families—known as 34-kDa and 32-kDa families—were identified in our *Psorophora* proteome; members of this family were described as immunogenic in the proteome study of *Ae. aegypti* saliva [43]. Also, the antigen-5 protein was confirmed in the *Psorophora* proteome, and members of this family have been previously described as a SG-secreted product in *Culex*[44]. Many of the identified proteins have homologs/orthologs in other mosquitoes that have been described as related to blood feeding.

### Insight into the *P. albipes* Secreted Sialome

The following highlights are related to the secreted sialome of *P. albipes* compared with others from bloodsucking Nematocera.

### Ubiquitous protein families

#### Enzymes

ʹ-nucleotidase/apyrases, adenosine deaminase, ribonuclease, endonuclease, alkaline phosphatase, serine proteases, lipase, destabilase/lysozyme, hyaluronidase, and glycosidases were identified. Cathepsins and serine-type carboxypeptidase are also noted but could be of H functions. These enzymes have all been found before in mosquito sialotranscriptomes, and their role in blood and sugar feeding has been reviewed [24]. Notably in the case of *Psorophora*, however, is the finding of both endonuclease (identified by MS/MS in band 12) and hyaluronidase, which were previously restricted to *C. quinquefasciatus*[24] and sand flies, but not found in *Aedes* or *Anopheles* sialotranscriptomes*.* This enzyme combination may help decrease skin-matrix viscosity and diffusion of salivary components, as well as breaking down neutrophil extracellular traps [45]. Apyrase, adenosine deaminase, and glycosidases were found by MS/MS in fraction 10, consistent with their expected sizes. Transcripts encoding for sphingomyelin phosphodiesterases (SMases)—some of which are highly transcribed with coverages higher than 500—is an unusual finding in mosquito sialotranscriptomes. Although lacking the initial methionine, Psor-15064 matches at position 6 a *C. quinquefasciatus* protein with 55% identity over 564 amino acids that has a predicted signal peptide. The SMases are members of the DNase I superfamily of enzymes responsible for breaking sphingomyelin into phosphocholine and ceramide. In addition, activation of SMase is suggested to play a role in production of ceramide in response to cellular stresses [46]. Tryptic peptides originating from SMase were found in fractions 11 and 12 of the NuPage gel in our proteomic analysis. The high expression of this enzyme suggests it may be secreted.

**Figure 2:**
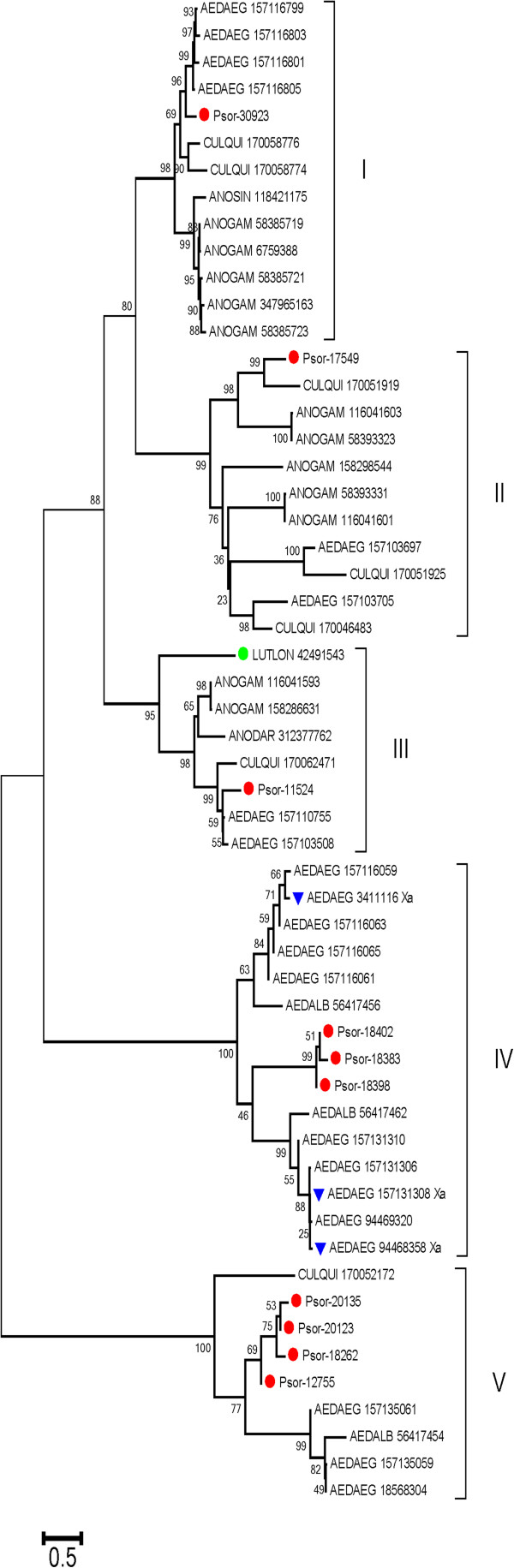
**Phylogram of the salivary serpins of *Psorophora albipes*.** The bootstrapped (1,000 iterations) phylogram was obtained using the serpins from *P. albipes* and their best-matching proteins from the non-redundant database from NCBI. *P. albipes* proteins are recognized by the prefix Psor- and a red marker (red circle symbol). Other proteins are represented by the first three letters of the genus name, followed by the first three letters of the species name, followed by their gi| accession number. The *Aedes aegypti* proteins identified as anti Xa anticlotting are marked with a blue symbol (blue reversed triangle symbol). The sole *Lutzomyia longipalpis* protein is identified by a green symbol (green circle symbol).The numbers at the nodes represent bootstrap support for 1,000 iterations using the neighbor-joining algorithm. The bar at the bottom indicates 50% amino acid substitution. Roman numerals indicate individual clades with strong bootstrap support.

*Protease inhibitor domains*: Serpins were well expressed, with 2.24% of the reads of the S class, and were identified by MS/MS in gel fraction 12. The protein encoded by Psor-18383 is 44% identical with the FXa-directed anticoagulant precursor of *Aedes albopictus*. Phylogenetic analysis indicated the presence of at least five distinct gene families (Roman numerals in Figure 2), of which clades I, II, and III are found in both culicines and anophelines (clade III also having a sand fly member), but clades IV and V are exclusively Aedine; clade IV includes the salivary Xase clotting inhibitor of *Ae. aegypti*[47]*.* The targets of serpins from clades I, II, III, and V remain to be identified. It is to be noted that the salivary anticlotting of anophelines is not a serpin but rather a novel protein family of antithrombins [48,49]. TIL and Kazal domain-containing peptides may be related to additional anticlotting proteins [50] or antimicrobials. A metalloproteinase inhibitor represents the first such finding in Nematocera sialomes; Psor-25577 is 85% and 78% identical to their *Ae. aegypti* and *C. quinquefasciatus* homologs, respectively. Psor-21372 codes for a pacifastin homolog, which may be an H protein. A poorly expressed cystatin may also be an H protein, but tick salivary cystatins are secreted and poorly expressed and could have immunosuppressive function [51,52].

#### Immunity-related proteins

Lysozyme, gambicin, cecropin, and defensins were found among antimicrobial agents. Pathogen recognition proteins of the ML domain, Fred/ficolin, Gram negative binding, peptidoglycan recognition, leucine-rich, galectin, and C-type lectin families were identified. Of these, lysozyme was identified in gel fraction 19 by MS/MS.

#### Yellow protein family

The yellow gene in *Drosophila* is responsible for tanning of the cuticle, and the mosquito homolog was shown to have a dopachrome oxidase function [53,54]. This protein family is specific to insects, the royal jelly protein being a member of the superfamily [55]. Interestingly, sand flies—but no other insect sialotranscriptomes—have two members of this family recently shown to be a scavenger of serotonin [56-58]. The *P. albipes* sialotranscriptome revealed two members of this family, probably alleles, relatively well expressed, assembled with over 200 × coverage. This is the first description of a yellow family member in mosquito sialotranscriptomes; however, these results derive from a high-coverage mosquito sialotranscriptome, and it may be possible that members of this family may be found in species previously studied if higher transcript coverage is attained.

### Mosquito-specific protein families

1,319,744 reads (18% of the total reads classified as S products) mapped to transcripts encoding proteins that can be classified according to their sequence similarity to 18 different protein families (21.68% of the total S protein families described in this transcriptome) previously described as unique to mosquitoes, i.e., they are not recognized in any other organism apart from mosquitoes [24]. A total of 69.23% of these mosquito-specific contigs had their best matches originating from *Aedes*, followed by 8.15% best matching to *Ochlerotatus,* 6.52% to *Culex*, and 4.66% to *Anopheles*. A previous review of Nematocera sialomes [24] proposed that some of these mosquito-specific families appear to be spread in all mosquito genera (studied so far), while others show specific distributions to a certain mosquito subfamily and/or genus. Accordingly, we conceptually divided our discussion regarding the mosquito-specific protein families present in *Psorophora* sialomes into four groups: *i*) mosquito-specific protein families common to Culicines and Anophelines, *ii*) mosquito-specific protein families thus far found only in Culicines, iii) mosquito-specific protein families unique to *Aedes/Ochlerotatus*, and *iv*) mosquito-specific protein families unique to *Culex*.

#### Mosquito-specific protein families common to Culicines and Anophelines

Nine of the 12 protein families previously known as common to Culicine and Anopheline were described in the *P. albipes* transcriptome: the HHH peptide family, the HHH peptide family 2, the mosquito basic tail family, the salivary protein 16 family, the *Aedes/Anopheles darlingi* 14-15 family, the gSG8 family, the Hyp6.2 family, the *Aedes* 62 kDa family, and the Anopheline SG1 family. Although commonly found in mosquito SG transcriptome analyses, no member of these families has been functionally characterized so far. Moreover, studies based on RT-PCR have assigned to some of these family members a tissue and/or sex specificity in their expression that suggests a role in the physiology of *Ae. albopictus* SGs [24].

**Figure 3:**
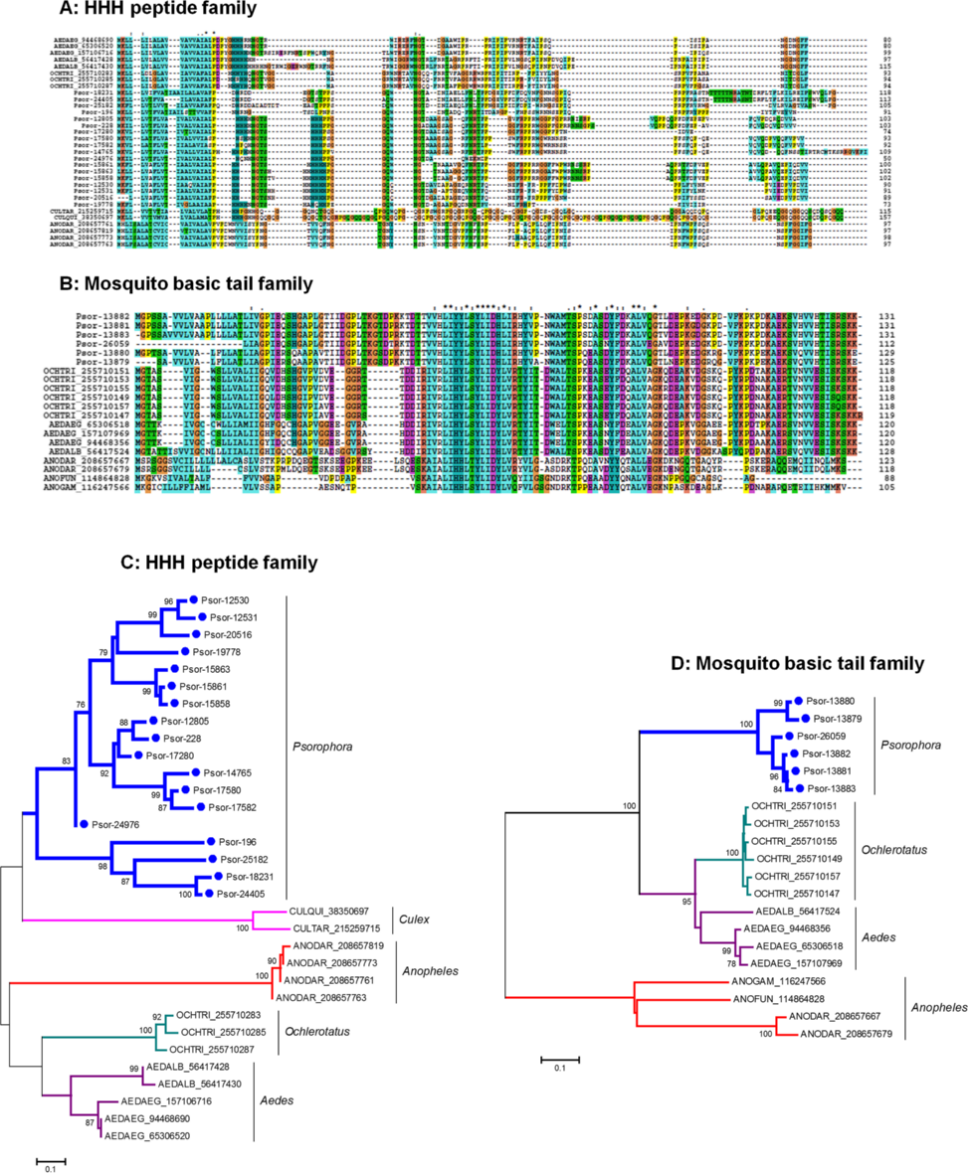
**Phylogram of salivary protein families derived from *Psorophora albipes *sialotranscriptome commonly found in Culicine and Anopheline mosquito sialotranscriptomes. A**, **C:** HHH peptide family. **B**, **D:** Mosquito basic tail family. Clustal alignment (**A** and **B**) and dendrogram (**C** and **D**) of all HHH peptide family and mosquito basic tail family, respectively, derived from the *Psorophora* sialotranscriptome. The symbols above the alignment indicate (*) identical sites, (:) conserved sites, and (.) less-conserved sites. The phylogram derived from the alignment of *Psorophora* proteins (indicate by Psor- and its contig number) with their best matches in the non-redundant database. The three first letters indicate the genus name from which each protein originates, followed by the three first letters of the species name, followed by the NCBI accession number. The numbers on the tree bifurcations indicate the percentage of bootstrap support above 75%. The scale bar at the bottom represents 10% amino acid substitution. Sequences were aligned by the ClustalW program, and the phylogram was made with the Mega package after 10,000 bootstraps with the neighbor-joining algorithm.

Among them, the HHH peptide family was previously suggested to play a role in antimicrobial defense because of its His richness as Zn ion chelators [24,59]. Here, this family was revealed as the fourth most abundantly expressed, with 7.65% of the total reads (Table 3). This family appears to be expanded in *Psorophora*, with a possible total of at least six genes (Figure 3). The abundant expression of this protein family suggests this protein as a good candidate for exposure marker to mosquito bites. Alignment of *Psorophora* transcripts encode two HHH repeats separated by NGTS amino acids, while one repeat was seen in the homologs from *Aedes, Ochlerotatus*, and *Culex* (Figure 3A); 35% to 55% identity is observed to the *Ae. aegypti* and *Ae. albopictus* homologs. The phylogram obtained after alignment of all HHH peptide genes found in mosquitoes shows at least five distinct clades with strong bootstrap support (Figure 3C). Two clades contain solely *Psorophora* transcripts (in several subclades). The remaining clades are specific to *Culex, An. darlingi*, *Ochlerotatus*, and *Aedes* (Figure 3C).

Mosquito basic tail proteins contain a Lys dipeptide tail (Figure 3B) and have been suggested as binding to negatively charged phospholipids found in cell membranes such as in the surface of platelets [60]. They may also be associated with plasminogen activation [61,62]. In the *Psorophora* transcriptome, six contigs (0.43% of the total contigs classified as S products) match mosquito basic tail peptides with 50% identity to *Ae. albopictus* family members (Additional file 1). Three tryptic peptides in our proteome analysis match contig Psor-13880, which encodes for a member of this family. Phylogenetic analysis of the basic tail mosquito family supports divergence of Culicine salivary proteins from the Anopheline family members (Figure 3D) where Anopheline and Culicine proteins are grouped in distinct clades (Figure 3D). Although Anophelines lack the basic tail, they have a conserved backbone (Figure 3B). In the Culicine clade, we observe that all *Psorophora* proteins are isolated in a genus-specific branch, separated from the other Culicine proteins with strong bootstrap support (Figure 3D).

Family Hyp6.2, represented with three truncated-sequences, is approximately 45% identical to the homologs from *Ochlerotatus* (Additional file 1). Additionally, all the contigs found in *P. albipes* transcriptome from the mosquito-specific families HHH family-2, salivary protein 16 family, *Aedes/An. darlingi* family, gSG8 family, and *Aedes* 62-kDa family have as their best matches the homologs from *Ae. aegypti*, with identities varying from 80% to 42% (Additional file 1)*.* Proteome analysis revealed tryptic peptides originating from *Psorophora* family members showing higher similarities to the *Aedes* 62-kDa family (Additional file 1).

#### Mosquito-specific protein families thus far found only in Culicines

To date, five protein families found in the *P. albipes* sialotranscriptome are unique to Culicines. Two of these (9.7-kDa and Hyp8.2 Culicine protein families) may play a role in blood feeding, as they are abundantly expressed in female *Ae. albopictus* SGs [63]. The 30.5-kDa and 23.5-kDa protein families appear to be involved in mosquito sugar feeding due to their reported expression in male and female SGs [63]; however, the tissue specificity of the fifth protein family—namely, the GQ-rich Culicine family—is still unknown [63,64]. So far, no member from these families has been functionally characterized.

Two abundantly expressed families in our transcriptome analysis are represented by the 30.5-kDa (4.35% of total reads encoding for S products) and Hyp8.2 Culicine families (1.30% of total reads encoding for S products). The first family was also within the 50 most-expressed families in this transcriptome (Table 3). Expression of these two families in *Psorophora* SGs was confirmed by our proteome analysis (Figure 1 and Table 4). Overall, they share 53% amino acid identity with the family member from *Ae. albopictus* (Additional file 1)*.* The *Psorophora* 9.7-kDa and 23.5-kDa families had *Ae. aegypti* proteins as their best BLAST similarity matches; tryptic peptides were found in our proteome analysis identifying these family members. In contrast, members of the GQ-rich Culicine family revealed 58% identity to its homologs from *C. quinquefasciatus* (Additional file 1).

**Figure 4:**
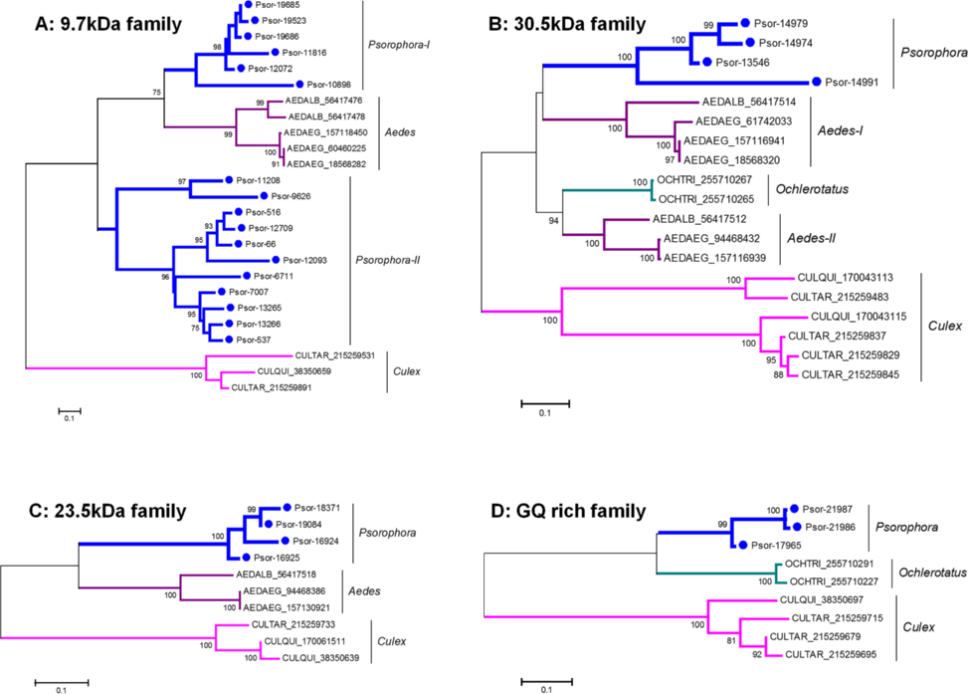
**Phylogram of salivary protein families derived from *Psorophora albipes *sialotranscriptome that are exclusively found in Culicine mosquitoes. A:** 9.7-kDa family. **B:** 30.5-kDa family. **C:** 23.5-kDa family. **D:** GQ-rich family. The phylogram derives from the alignment of *Psorophora* proteins (indicated by Psor- and their contig number) and their comparison with their best matches in the non-redundant database. The three first letters indicate the genus name, followed by the three first letters of the species name, followed by the NCBI accession numbers. Numbers on the tree bifurcations indicate the percentage of bootstrap support above 75%. The bar at the bottom represents 10% amino acid substitution. Scale sequences were aligned by the ClustalW program, and the phylogram was made with the Mega package after 10,000 bootstraps with the neighbor-joining algorithm.

Here we performed phylogenetic analysis of the 9.7-kDa family (Figure 4A), the 30.5-kDa family (Figure 4B), the 23.5-kDa family (Figure 4C), and GQ-rich family (Figure 4D). Overall, all four phylograms show *Psorophora* proteins phylogenetically far from *Culex* proteins. The phylogenetic tree of the 9.7-kDa family (Figure 4A) shows at least four different transcript clusters in *Psorophora* (one cluster in the branch named *Psorophora*-I and three in *Psorophora*-II). Also, several gene duplications can be found in each cluster. This phylogeny shows that *Aedes* family members are closer to *Psorophora* family members, while *Culex* proteins appear as an outgroup (Figure 4A).

The phylogram of the 30.5-kDa (Figure 4B) and 23.5-kDa (Figure 4C) families confirm the same pattern seen for the 9.7-kDa family (Figure 4A) in the sense that *Psorophora* proteins are grouped in the same clade with *Aedes* proteins (Figure 4A–C). The GQ-rich family shows *Psorophora* members grouped within the same clade containing *Ochlerotarus* proteins (Figure 4D). Although previous studies using 18S rDNA sequence suggested *Psorophora* species as a sister group to *Culex* and/or a sister group to the *Aedes*/*Ochlerotatus* species [12,16], our results suggest—based on the composition of the salivary proteins—that *Psorophora* is much closer to *Aedes* than to *Culex*.

#### Mosquito-specific protein families unique to Aedes/Ochlerotatus mosquitoes

**Figure 5:**
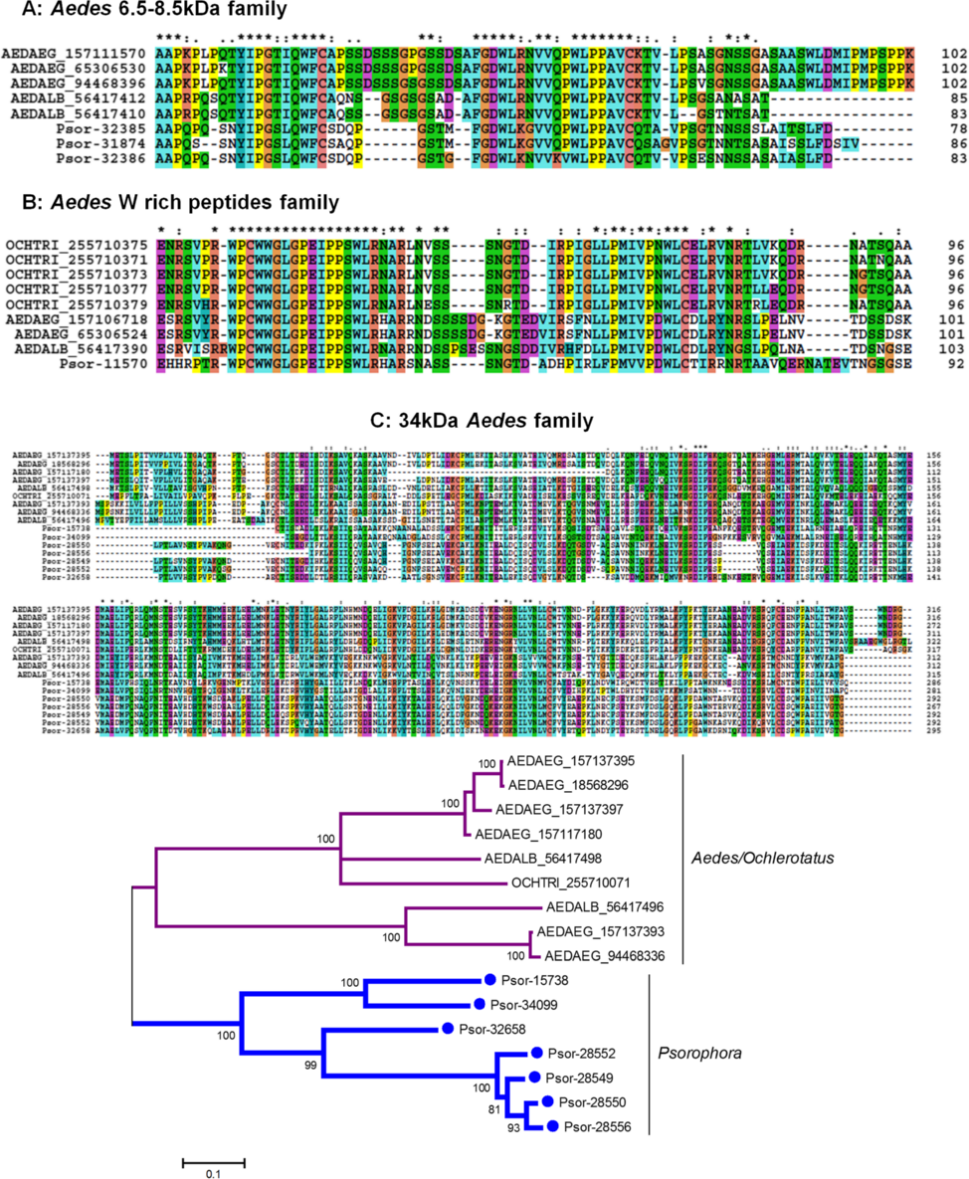
**Phylogenetic analyses of salivary *Psorophora *protein families previously found exclusively in *Aedes/Ochlerotatus *mosquitoes.** Clustal alignment of all *Aedes* 6.5–8.5-kDa proteins **(A)** and *Aedes* W-rich peptides **(B)** derived from *Psorophora* sialotranscriptome. Symbols above the alignment indicate (*) identical sites, (:) conserved sites, and (.) less-conserved sites. Aedes 34-kDa family alignment and bootstrapped phylogram **(C)**. The numbers on the tree bifurcations indicate the percentage bootstrap support above 75%. The scale bar at the bottom represents 10% amino acid substitution. Sequences were aligned by the ClustalW program, and the dendrogram was made with the Mega package after 10,000 bootstraps with the neighbor-joining algorithm.

Three protein families—*Aedes* 6.5–8.5-kDa family (Figure 5A), *Aedes* W-rich peptides family (Figure 5B), and *Aedes* 34-kDa family (Figure 5C)—previously described as exclusive to *Aedes/Ochlerotatus* were found in the *Psorophora* genus. Previous studies showed that these families are female- and SG-specific [63,64], but their function still remains unknown. Alignment of the transcripts found in *Psorophora* encoding for *Aedes* 6.5–8.5-kDa (Figure 5A) and *Aedes* W-rich peptides (Figure 5B) families reveal higher identity in their amino acid sequences to *Ae. albopictus* (55%) and to *Ochlerotatus triseriatus* (62%), respectively (Additional file 1). Here, only the 34-kDa family was confirmed as present in the *Psorophora* SG proteome (fraction F14; Figure 1, Table 4). Alignments of 34-kDa family members showed 29–37% identity to their homologous proteins from *Aedes* mosquitoes (Figure 5C and Additional file 1). The phylogram shows all *Psorophora* proteins of the 34-kDa family grouped in the same clade, while the second clade of the phylogram contains all the *Aedes/Ochlerotatus* gene products (Figure 5C).

#### Protein family so far found only in Culex

**Figure 6:**
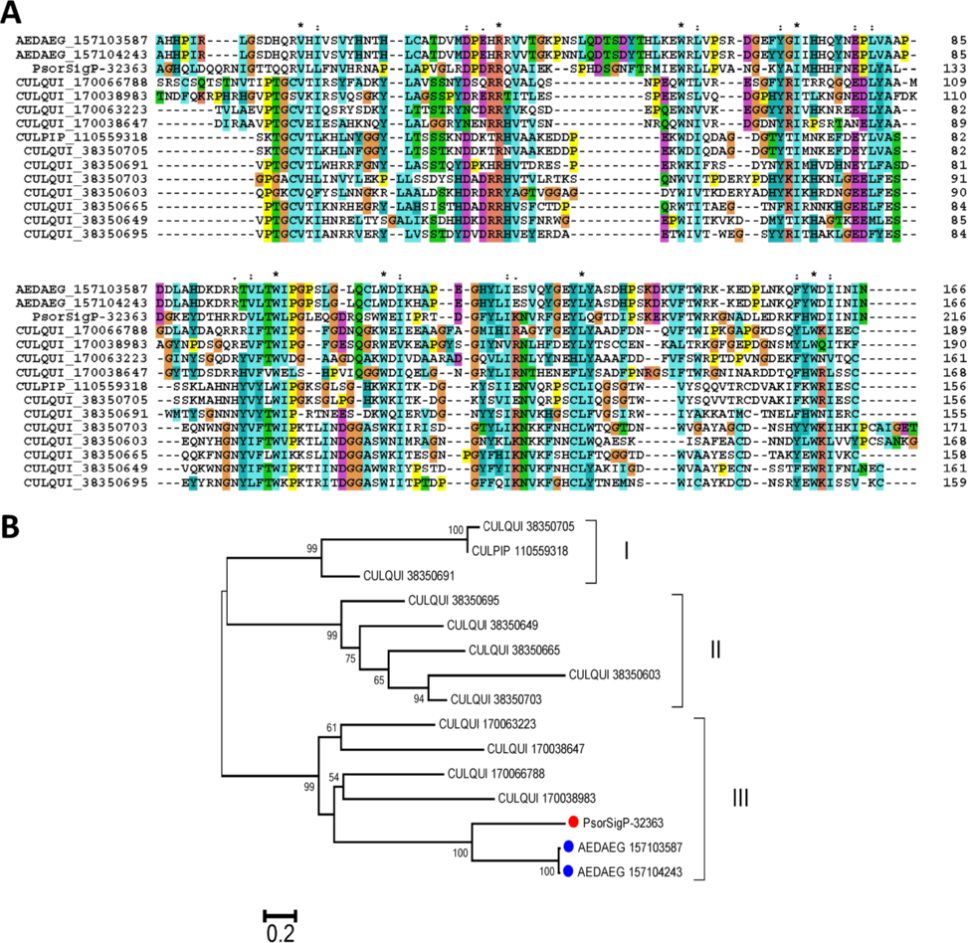
**Mosquito proteins of the W-rich peptides/16-kDa protein family of *Culex. *(A)** Clustal alignment. Symbols above the alignment indicate (*) identical sites, (:) conserved sites, and (.) less-conserved sites. **(B)** Bootstrapped phylogram derived from the alignment in **A**. Numbers on the tree bifurcations indicate the percentage bootstrap support above 50%. The scale bar at the bottom represents 20% amino acid substitution. Sequences were aligned by the ClustalW program, and the dendrogram was made with the Mega package after 1,000 bootstraps with the neighbor-joining algorithm. The *Psorophora albipes* proteins are recognized by the prefix Psor- and a red marker (red circle symbol). Aedes proteins are indicated with a blue marker (blue circle symbol). Other proteins are represented by the first three letters of the genus name, followed by the first three letters of the species name, followed by the gi| accession number. Roman numerals indicate individual clades with strong bootstrap support.

The *Culex* W-rich protein (WRP)/16-kDa family is a salivary protein family so far uniquely found in *Culex* sialotranscriptomes, where nearly 20 genes coding for this family are known, subdivided into different subfamilies varying in their number of cysteine residues [44,65]. Although highly expressed and specific to *Culex,* the function of the WRP/16-kDa family remains still unclear. Here we report for the first time members of this family originating from a non-*Culex* mosquito. A total of 2,138 reads were found grouped into two contigs, Psor-32363 and Psor-32364, the latter being a truncated variant of the first with a few amino acid changes. The mature MW of the encoded polypeptides is approximately 24 kDa with an estimated isoelectric point of 7.1 and amino acid sequences that are W rich (Additional file 1). Interestingly, the *Psorophora* protein best matches two putative *Ae. aegypti* proteins never previously described in sialotranscriptomes. Alignment of the *P. albipes* sequence with *Aedes* and selected *Culex* sequences shows three conserved tryptophan residues among a total of 8 identities and 22 similarities, with a total similarity of only 14% (Figure 6A). Phylogenetic analysis (Figure 6B) groups the *Psorophora* and *Aedes* sequences with 100% bootstrap support within a clade of four *Culex* proteins having 99% bootstrap support (Clade III in Figure 6B). These results suggest that Culicines shared a common ancestor of a gene coding for this protein family that expanded in *Culex* but not in *Aedes*, indicating this family was not a *Culex* “invention.” The *Psorophora* member of the family thus helped us to partially understand the evolution of this family in *Culex* by providing a link between *Culex* and *Aedes* sequences*.*

### Other putative secreted proteins

Two putative S protein sequences match black fly proteins previously thought to be unique to *Simulium* sialomes. Three previously thought to be orphan proteins of *Aedes* and *Ochlerotatus* (*Aedes* 7-kDa and 5-kDa families and *Ochlerotatus* OT-19 family) were deorphanized. Eight novel salivary protein families were found in the *Psorophora* sialotranscriptome, four of which appear unique to *Psorophora*, while the others have matches to mosquito hypothetical proteins not previously described in sialotranscriptomes.

We additionally identified 372 transcripts sequences encoding for secreted polypeptides, most of which have no relevant matches to any sequence deposited thus far in the NR database. Two of these were identified by proteome analysis. All details of these proteins are in the hyperlinked Excel spreadsheet available in Additional file 1.

### *P. albipes* similarities to other mosquito species

**Figure 7:**
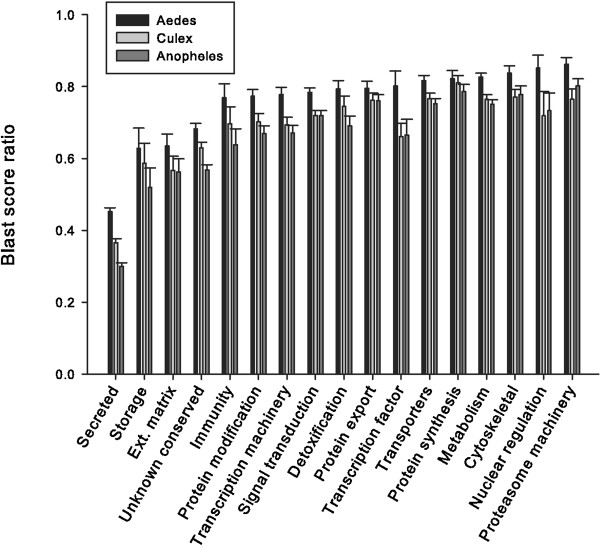
**Comparison of *Psorophora albipes *protein sequences to those of *Aedes aegypti (*black square symbol*), Culex quinquefasciatus (*dark gray symbol*)*, and *Anopheles gambiae *(SlateGray square symbol) by blastp score ratio.** The bars and lines represent the average ratio and standard error calculated by dividing the score of the best-matching mosquito protein and the self-score of the protein for the functional categories indicated in the abscissa. For details, see text.

The availability of the genomes of *Ae. aegypti, C. quinquefasciatus,* and *Anopheles gambiae*[66-68] allows for comparisons of the protein sequences of *Psorophora* to those deducted from the three mosquito genomes. We have determined (Additional file 1) the BLAST score ratios of each protein for the three genomes by dividing the BLAST score found for the blastp result against one of the three mosquito proteomes by the BLAST score of the *Psorophora* protein blasted against itself [39]. The comparisons indicate that *Aedes* is the closest related mosquito to *Psorophora*, followed by *Culex* and *Anopheles* (Figure 7). It also shows that the S class of proteins has the lowest ratio of all, while those for proteasome machinery, nuclear regulation, and cytoskeletal are among the most conserved (Figure 7). In the S class computed above, those 372 proteins indicated as “Other putative secreted peptides” were not included, as they are of uncertain nature and would further decrease the score ratios. This divergence of salivary proteins in mosquitoes has been previously reported for other taxa [22,24,63-65,69-71].

### Polymorphism of *P. albipes* coding sequences inferred from the RNAseq data

**Figure 8:**
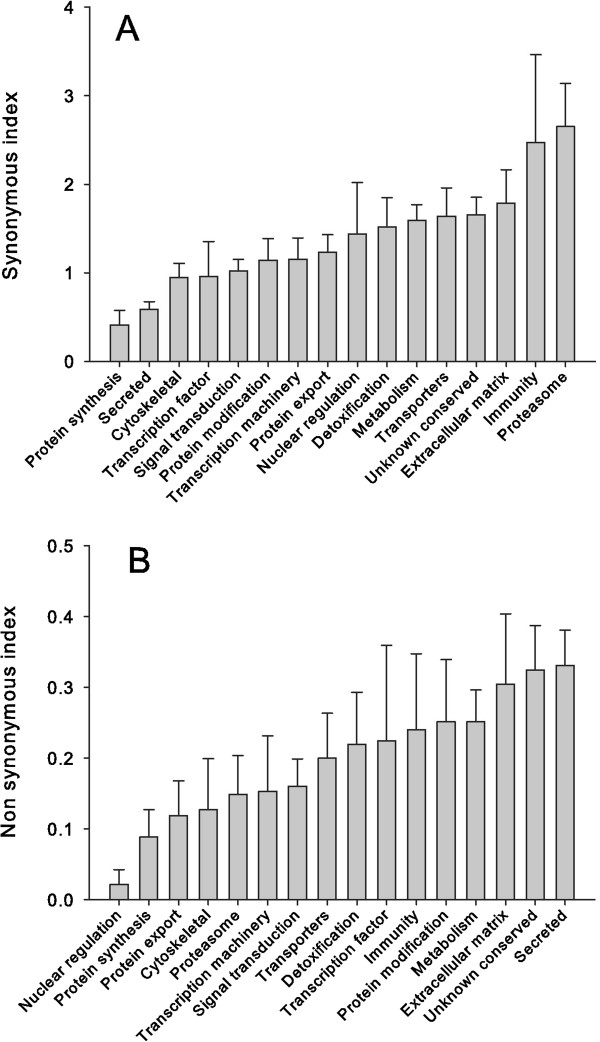
**Number of polymorphic sites per 100 codons in *Psorophora albipes *proteins.** Bars represent the average and standard errors of synonymous **(A)** and non-synonymous **(B)** sites per 100 codons in the indicated functional categories.

RNAseq data produces high contig coverage. In our CDS set, 1,822 had average coverage of 100 or larger per nucleotide site, allowing reliable identification of SNPs using the tools BWA and Samtools (see Methods). From this CDS set we excluded the 372 proteins indicated in the previous section, plus all of the unknown class, and all CDS having a similar protein sequence with > 95% similarity (to avoid overrepresentation of alleles), producing a set of 1,100 CDS. When comparing the number of synonymous sites per 100 codons among different functional classes, the protein syntheses and secreted classes have the smallest value, while the proteasome and immune categories have the highest (Figure 8A and Table 5). When the values of the number of non-synonymous SNPs are compared, the figure reverses (Figure 8B), the secreted category having the highest value, to near 0.33 per 100 codons (Table 5). The overall non-synonymous to synonymous rate also shows the secreted class to have the highest ratio (Table 5). This increased non-synonymous polymorphism is not an artifact resulting of increased read coverage of the contigs of the secreted class because the protein synthesis class of contigs has an even higher read coverage but has the second smallest non-synonymous polymorphism index. It is possible that this high value of non-synonymous polymorphism observed for the secreted class may result from chimeric assembly of coding sequences originating from multiple recently duplicated genes coding for very similar proteins. At any rate, this high polymorphism may underlie the mechanisms leading to accelerated evolution of salivary proteins observed in bloodsucking arthropods.

**Table 5:** ***Psorophora albipes *** **polymorphisms detected on a set of 1,100 coding sequences of 16 functional classes**

**Class**	**Average (Syn /Codon) x 100**^**1**^	**SE**	**Average (NS /codon) x 100**^**2**^	**SE**	**NS/Syn**	**Average coverage**^**3**^	**SE**	**N**
Nuclear regulation	1.4343	0.5854	0.0217	0.0207	0.0152	278.6	48.3	11
Protein synthesis	0.4095	0.1637	0.0885	0.0389	0.2161	2981.7	243.9	96
Protein export	1.2332	0.1954	0.1188	0.0491	0.0963	340.0	34.0	65
Cytoskeletal	0.9455	0.1606	0.1271	0.0722	0.1344	322.5	48.0	54
Proteasome	2.6511	0.4884	0.1487	0.0550	0.0561	189.0	20.4	35
Transcription	1.1524	0.2376	0.1531	0.0785	0.1329	410.2	99.3	47
Signal transduction	1.0220	0.1283	0.1604	0.0385	0.1570	365.9	69.9	143
Transporters	1.6399	0.3186	0.1997	0.0638	0.1218	400.2	61.0	89
Detoxification	1.5145	0.3318	0.2194	0.0739	0.1448	198.5	31.2	16
Transcription factor	0.9573	0.3958	0.2243	0.1351	0.2343	237.2	30.2	12
Immunity	2.4690	0.9928	0.2398	0.1077	0.0971	224.7	47.6	11
Protein modification	1.1421	0.2421	0.2514	0.0877	0.2201	370.1	86.2	56
Metabolism	1.5898	0.1807	0.2515	0.0447	0.1582	389.2	72.1	147
Extracellular matrix	1.7871	0.3776	0.3044	0.0994	0.1703	280.6	39.9	29
Unknown conserved	1.6564	0.1961	0.3244	0.0626	0.1958	372.9	44.6	127
Secreted	0.5885	0.0849	0.3308	0.0498	0.5621	2729.7	288.8	334
Total								1100

^1^Number of synonymous polymorphism per 100 codons.

^2^Number of non-synonymous polymorphism per 100 codons.

^3^Average read coverage of coding sequences.

## Conclusions

The sialotranscriptome of *P. albipes* as described here is the first—or among the first—to use solely Illumina sequences for its assembly, in the absence of a reference genome. Over 3,000 coding sequences were recovered, 1,790 of which were submitted to GenBank. This is also the first transcriptome of a member of the *Psorophora* genus. As expected, the protein sequences presented more similarities to *Aedes*, followed by *Culex* and *Anopheles* proteins. Despite this more Aedine nature, *P. albipes* presented some *Culex* characters—such as the presence of endonuclease and hyaluronidase—common in sand flies and black flies but so far uniquely found in *Culex*. A *Psorophora* protein similar to the WRP/16-kDa family also unique so far to *Culex* allowed the discovery of a “missing link” between this *Culex* family and hypothetical *Ae. aegypti* proteins, indicating this gene family is ancestral in all Culicines but poorly or not expressed in *Aedes* SGs. Orphan protein families from *Aedes* and *Ochlerotatus* were deorphanized, and several new families of proteins were identified, four of which appear unique to *Psorophora*, supporting the idea that sialotranscriptomes of new bloodsucking genera yield at least two novel protein families [72]. However, these novel sequences may result from misassemblies or chymeras. Further sequencing of other *Psorophora* species may clarify this area. Unique to *Psorophora* is also the finding of SMase, not previously found in mosquito sialomes. Because the sample derived from 50 field-collected mosquitoes, we also were able to derive an estimate of SNPs and the rate of synonymous and non-synonymous mutations in this data set.

### Availability of supporting data

All data from the transcriptome and proteome analysis of *P. albipes* SGs are disclosed in Additional file 1, a hyperlinked Excel spreadsheet available at http://exon.niaid.nih.gov/transcriptome/Psorophora_albipes/Pso-s2-web.xlsx. Raw reads were deposited in the SRA of the NCBI under bioproject numbers PRJNA208524 and 208958 and raw data file SRR908278. One thousand seven hundred and ninety coding sequences have been publicly deposited in the Transcriptome Shotgun Assembly project at DDBJ/EMBL/GenBank under accession GALA00000000. The version described in this paper is the first version, GALA01000000, ranging from GALA01000001 to GALA01001790.

## Abbreviations

ABySS: Assembly by short sequences software; bcftools: Binary call format tools; CDS: Coding sequences; H: Housekeeping proteins; MS: Mass spectrometry; MS/MS: Tandem MS; MW: Molecular weight; NCBI: National Center for Biotechnology Information; NGS: Next-generation sequencing; NISC: NIH Intramural Sequencing Center; S: Secreted proteins; samtools: Sequence alignment/map tools; SG: Salivary gland; SMase: Sphingomyelin phosphodiesterase; SNP: Single-nucleotide polymorphism; SRA: Sequence read archives; U: Proteins of unknown function; WRP: W-rich protein.

## Competing interests

The authors declare that they have no competing interests.

## Authors’ contributions

ACC, EC, and JMCR contributed to experimental design, bioinformatics analysis, and writing of the manuscript. CMRV, FACP, and JFM contributed to insect collection, dissections, and taxonomic identification of mosquitoes. All authors read and approved the final manuscript.

## Supplementary Material

Additional file 1 Hyperlinked Excel spreadsheet with protein coding sequences and added information. Click here for file

Additional file 2 Assembly information. Click here for file
