# Supplementary material for: A deep insight into the sialotranscriptome of the mosquito, Psorophora albipes
Source: BMC Genomics. 2013 Dec 13;14:875. doi: 10.1186/1471-2164-14-875 (PMC3878727; doi:10.1186/1471-2164-14-875)
Supplement: Additional file 2 — Assembly information. [file 1471-2164-14-875-S2.docx]

Supplemental file 2:

Assembly characteristics of 135,651,020 reads of length = 101 nucleotides. For details, see methods.

| **Contig size** | **No. of sequences** | **No. of bases** |
| --- | --- | --- |
| <100 | 1 | 63 |
| 101-150 | 9,424 | 1,249,997 |
| 151-200 | 6,504 | 1,112,729 |
| 201-300 | 2,028 | 487,167 |
| 301-400 | 1,275 | 444,490 |
| 401-500 | 1,264 | 571,999 |
| 501-750 | 4,751 | 2,969,661 |
| 751-1000 | 4,234 | 3,688,816 |
| 1001-2000 | 9,497 | 13,330,048 |
| 2001-3000 | 3,087 | 7,436,888 |
| 3001-5000 | 1,355 | 4,972,308 |
| 5001-10000 | 324 | 2,027,471 |
| 10001-15000 | 15 | 172,313 |
| > 1500 | 7 | 115,827 |
|  |  |  |
| Total | 43,766 | 38,579,777 |
| L50 | 573 |  |

| **Raw number of bases** | **Assembled number of bases** | **Compaction** |
| --- | --- | --- |
| 13,700,753,020 | 38,579,777 | 355.12784 |

From the above assembly, 3,247 coding sequences (CDS) were extracted with the following characteristics:

| **CDS size** | **No. of sequences** | **No. of bases** |
| --- | --- | --- |
| 151-200 | 442 | 76,320 |
| 201-300 | 522 | 128,097 |
| 301-400 | 397 | 137,313 |
| 401-500 | 268 | 121,284 |
| 501-1000 | 952 | 683,298 |
| 1001-2000 | 576 | 777,759 |
| 2001-3000 | 62 | 147,987 |
| 3001-5000 | 24 | 83,691 |
| 5001-10000 | 4 | 25,536 |
|  |  |  |
| Total | 3,247 | 2,181,285 |

The 3,247 CDS mapped 13,535,229 reads with the following characteristics:

|  | **Number of mapped reads** | **Average CDS coverage per base** |
| --- | --- | --- |
| Average | 4,168.5 | 614.9 |
| Max | 742,884 | 29,855.4 |
| Min | 10 | 4.2 |
| Total | 13,535,229 | - |
